# Reorganisation of GP surgeries during the COVID-19 outbreak: analysis of guidelines from 15 countries

**DOI:** 10.1186/s12875-021-01413-z

**Published:** 2021-05-17

**Authors:** Rosy Tsopra, Paul Frappe, Sven Streit, Ana Luisa Neves, Persijn J. Honkoop, Ana Belen Espinosa-Gonzalez, Berk Geroğlu, Tobias Jahr, Heidrun Lingner, Katarzyna Nessler, Gabriella Pesolillo, Øyvind Stople Sivertsen, Hans Thulesius, Raluca Zoitanu, Anita Burgun, Shérazade Kinouani

**Affiliations:** 1 INSERM, Université de Paris, Sorbonne Université, Centre de Recherche des Cordeliers, Information Sciences to support Personalized Medicine, F-75006 Paris, France; 2 Department of Medical Informatics, Hôpital Européen Georges-Pompidou, AP-HP, Paris, France; 3 Department of general practice, Faculty of medicine Jacques Lisfranc, University of Lyon, Saint-Etienne, France; 4 Inserm UMR 1059, Sainbiose DVH, University of Lyon, Saint-Etienne, France; 5 Inserm CIC-EC 1408, University of Lyon, Saint-Etienne, France; 6 College of General Practice / Collège de la Médecine Générale, Paris, France; 7 Institute of Primary Health Care (BIHAM), University of Bern, Bern, Switzerland; 8 Patient Safety Translational Research Centre, Institute of Global Health Innovation, Imperial College London, London, UK; 9 Center for Health Technology and Services Research / Department of Community Medicine, Health Information and Decision, Faculty of Medicine, University of Porto, Porto, Portugal; 10 Department of Public Health and Primary Care, Leiden University Medical Center, Leiden, The Netherlands; 11 Imperial College London, London, UK; 12 İzmir Karşıyaka District Health Directorate, İzmir, Turkey; 13 Medizinische Hochschule Hannover, OE 5430, Carl Neuberg Str. 1, 30625 Hannover, Germany; 14 Medizinische Hochschule Hannover, Medizinische Psychologie, OE 5430, Hannover, Germany; 15 Member of the German Center for Lung Research (DZL)/ BREATH - Biomedical Research in Endstage and Obstructive Lung Disease Hannover, Carl Neuberg Str. 1, 30625 Hannover, Germany; 16 Department of Family Medicine, Jagiellonian University Medical College, Kraków, Poland; 17 Vasco da Gama Movement, Wonca Europe, Kraków, Poland; 18 ASL Teramo, Abruzzo, Italy; 19 Torshovdalen Health Center, Oslo, Norway; 20 Editor of the Journal of the Norwegian Medical Association, Oslo, Norway; 21 Lund University and Linneaus University, Lund, Sweden; 22 National Federation of Family Medicine Employers in Romania (FNPMF), București, Romania; 23 Department of Medical Informatics, Hôpital Européen Georges-Pompidou & Necker Children’s Hospital, AP-HP, Paris, France; 24 INSERM, Bordeaux Population Health Research Center, team HEALTHY, UMR 1219, university of Bordeaux, F-33000 Bordeaux, France; 25 Department of General Practice, University of Bordeaux, 146 rue Léo Saignat, F-33000 Bordeaux, France

**Keywords:** COVID-19, General Practitioner, Primary care, Clinical Practice Guidelines, Pandemic

## Abstract

**Background:**

General practitioners (GPs) play a key role in managing the COVID-19 outbreak. However, they may encounter difficulties adapting their practices to the pandemic. We provide here an analysis of guidelines for the reorganisation of GP surgeries during the beginning of the pandemic from 15 countries.

**Methods:**

A network of GPs collaborated together in a three-step process: (i) identification of key recommendations of GP surgery reorganisation, according to WHO, CDC and health professional resources from health care facilities; (ii) collection of key recommendations included in the guidelines published in 15 countries; (iii) analysis, comparison and synthesis of the results.

**Results:**

Recommendations for the reorganisation of GP surgeries of four types were identified: (i) reorganisation of GP consultations (cancelation of non-urgent consultations, follow-up via e-consultations), (ii) reorganisation of GP surgeries (area partitioning, visual alerts and signs, strict hygiene measures), (iii) reorganisation of medical examinations by GPs (equipment, hygiene, partial clinical examinations, patient education), (iv) reorganisation of GP staff (equipment, management, meetings, collaboration with the local community).

**Conclusions:**

We provide here an analysis of guidelines for the reorganisation of GP surgeries during the beginning of the COVID-19 outbreak from 15 countries. These guidelines focus principally on clinical care, with less attention paid to staff management, and the area of epidemiological surveillance and research is largely neglected. The differences of guidelines between countries and the difficulty to apply them in routine care, highlight the need of advanced research in primary care. Thereby, primary care would be able to provide recommendations adapted to the real-world settings and with stronger evidence, which is especially necessary during pandemics.

**Supplementary Information:**

The online version contains supplementary material available at 10.1186/s12875-021-01413-z.

## Background

In December 2019, China declared its first cases of COVID-19 in Wuhan, Hubei Province [1]. The causal agent, SARS-CoV-2, then rapidly spread around the world, leading to the declaration of a pandemic by the World Health Organisation (WHO) on March 11^th^, 2020 [2]. All countries worldwide are now battling SARS-CoV-2 and dealing with the healthcare burden it represents [3]. The main challenge is “resisting” the spread of the virus and ensuring that emergency and intensive care units are not overwhelmed by massive surges in patient numbers.

General practitioners (GPs) are on the frontline in the battle against COVID-19 and have a key role to play in the management of the crisis. They are involved in the education, triage and diagnosis of patients [4, 5], and in screening for those at risk of developing severe symptoms. GPs can greatly alleviate the burden on hospital by dealing with non-severe forms of COVID-19, which account for most cases [6]. Given the risk of GP exposure to the virus [6–8] and of transmitting SARS-CoV-2 to vulnerable patients, it was crucial for GPs to adapt their surgeries and practices rapidly to the pandemic. This has led to the adoption of telemedicine [7, 9, 10]; the equipment of GPs with personal protective equipment (PPE); and the provision of masks to COVID-19 patients in some cases, to prevent the spread of the disease [7, 11]. However, some GPs, particularly those working in smaller practices, have encountered difficulties introducing such changes [12].

Several countries have developed national guidelines for primary care organisation to assist GPs. These guidelines highlight the need to reorganise GP surgeries for the safe management of patients with COVID-19, but also to provide continuity of care for all, regardless COVID-19 status [13]. However, the contents of the guidelines differ between countries, and some countries have yet to issue recommendations for primary care. We felt that it would be useful to have an overview of these guidelines, to facilitate decision-making in countries that have yet to establish guidelines, and for future reference, for country-by-country assessments of the impact of national initiatives on the course of the epidemic.

We therefore aim here to provide a multi-country analysis of the reorganisation of GP surgeries during the beginning of the COVID-19 outbreak, based on a comparison of guidelines published in 15 countries.

## Methods

A network of GPs from various countries, all belonging to the international GP organisation WONCA, collaborated in the following three-step protocol to identify similarities and differences in GP surgery reorganisation between their home countries:

- Step 1: Identification of an initial set of key recommendations for the reorganisation of GP surgeries

RT collected key items relating to GP surgery reorganisation from resources published by the WHO [14] and the Centers for Disease Control and Prevention (CDC) [15], and dedicated resources for healthcare workers at facility level. Key items were then attributed to a theme and grouped into categories.

SK then reviewed each category and item for accuracy and comprehensibility, and added new items based on experiences as healthcare professional from care facilities, in consensus with RT. This resulted in an initial set of key recommendations relating to GP surgery reorganisation (See Additional file 1).

- Step 2: Selection of guidelines and data collection

Voluntary GP-reviewers participated in this study between April 25^th^ and May 5^th^ 2020. They received an Excel® file with both instructions and the set of key recommendations gathered in step 1.

The GP-reviewers were first asked to identify the national guidelines published on the topic in their country. Ideally, these guidelines were issued by health authorities and/or GP national colleges. If this was not possible, the GP-reviewers were allowed to use any other source of guidance commonly used by GPs in their country (e.g. letters from GP colleges). For each guideline, each GP-reviewer specified the provider, the date of publication, and pandemic level (number of positive cases and deaths at the time of publication).

Each GP-reviewer then analysed the guidelines against the set of key recommendations identified in step 1, determining:

- Whether the key recommendations were found in national guidelines
- Whether there were new recommendations mentioned in the national guidelines but missing from the initial set of key recommendations.

Despite the lack of GP-reviewers from the United States of America within our network, we included the USA in this study, as the USA is currently the country with the largest numbers of cases and deaths. RT and SK collected USA data from other guidelines published by the CDC [13, 16] and the American Academy of Family Physicians [17].

- Step 3: Data analysis, comparison and synthesis

RT collected and compared the analyses provided by the GP-reviewers. New recommendations were mapped to existing categories, and if this was not possible, new categories were created. This resulted in a new set of categories and recommendations, which was then reviewed by each GP-reviewer. Potential changes were considered by RT for the final analysis.

## Results

Four types of GP surgery reorganisation were identified from the analysis of guidelines issued by 15 countries (see Additional file 2).

### Theme 1: Reorganisation of GP consultations (Table 1)

All countries recommended alternatives to face-to-face consultations, such as phone consultations or telemedicine (Table 1). In cases of face-to-face consultations, the guidelines recommended scheduling patient appointments to ensure a short waiting time, and re-scheduling appointments if the patient developed respiratory symptoms (due to COVID-19 or not). Dedicated time slots for COVID-19 patients should also be organised.

**Table 1:** Reorganisation of GP consultations

	WHO	France	Germany	Italy	Netherlands	New Zealand	Norway	Poland	Portugal	Romania	Spain	Sweden	Switzerland	Turkey	United Kingdom	US
**For all patients: Explore alternatives to face-to-face consultations**																
- Consultation by phone		x	x	x	x	x	x	x	x	x	x	x	x	x	x	x
- Consultation by telemedicine		x		x	x	x	x	x		x	x	x	x	x	x	x
- Dedicated phone centre (for information and/or medical advice)		x	x	x	x	x	x	x	x	x	x	x	x	x	x	
**For all patients: If face-to-face consultations occur**																
- Schedule consultations		x	x	x	x	x	x	x	x	x	x	x	x		x	
- Provide dedicated time slots for COVID-19 patients		x	x		x	x			x	x		x	x			
- Ensure a short waiting time			x	x	x		x	x		x		x	x			
- Reschedule if the patient develops respiratory symptoms			x	x		x	x	x	x	x	x	x			x	x
**For non-COVID-19 patients**																
- Postpone elective procedures and non-urgent outpatient visits		x	x	x	x	x	x	x	x	x	x	x	until 27/04	x	x	x
- Cancel group healthcare activities			x		x	x	x	x	x	x			until 27/04		x	x
- Maintain mandatory vaccinations							x	x	x	x			x	x	x	x
- Initiate telephone contact with the patient		x	x	x	x	x	x	x	x	x	x	x		x	x	
- Accept the renewal of drug prescriptions by pharmacists		x				x		x	x		x			x		
**For COVID-19 patients**																
- Remote consultation for initial diagnosis		x	x	x		x	x	x	x	x	x	x	x	x	x	
- Remote consultation for monitoring		x	x	x	x	x	x	x	x	x	x	x	x	x	x	
- Contact patient between days 6 and 8 for monitoring		x		x	x									x		
- Contact risk group patients daily									x			x				
- Contact patient until recovery									x					x		
- Develop assessment protocols for COVID-19 patients		x	x	x	x	x			x		x	x	x	x	x	x
- Develop protocols for rapid triage			x	x	x	x		x	x		x	x	x	x	x	x
- Online tools for patients		x			x		x	x	x		x			x	x	x
- Online tools for healthcare professionals		x	x		x		x	x	x			x	x	x	x	x

All countries recommended postponing non-urgent consultations and procedures, and a few authorized the renewal of drug prescriptions by pharmacists during the pandemic without a mandatory GP consultation. However, almost all countries also highlighted the need for GPs to remain in contact with patients with chronic diseases.

Almost all countries recommended remote consultations for the diagnosis and monitoring of COVID-19 patients (including suspected COVID-19 patients). The mode of monitoring differed between countries: daily for risk group patients in Sweden and Portugal, between days 6 and 8 for France, Italy, and Turkey, or until patient recovered in Portugal and Turkey. Most countries also recommended developing protocols for the rapid triage and assessment of COVID-19 patients. Eleven countries developed online tools for GPs, such as websites, webinars, or decision tools. Portugal implemented a national online platform accessible to GPs, for the tracing and monitoring of COVID-19 patients. Some countries also developed dedicated phone centres and online tools for patient information, such as chatbots accessible via Whatsapp® in Spain and Turkey.

### Theme 2: Reorganisation of GP surgeries (Table 2)

Recommendations for face-to-face triage stations differed between countries (Table 2). Germany, Italy, Poland, Romania, Spain, Switzerland, and the USA had the most detailed recommendations, whereas other countries, such as the UK recommended that all triage should be performed remotely, resulting in a lack of guidance concerning face-to-face triage. Triage stations should be located outside the facility, with a single point of entry and physical barriers. All patients should be screened, recorded and should wear a face mask or appropriate alternative. Seven countries also recommended that patients should be asked to wash their hands, and, in Switzerland, it was recommended that patients be asked not to touch doors and door handles. It was also recommended that the number of people accompanying the patient should be limited and, in Spain, that these individuals should also wear face masks. Hygiene supplies and visuals alerts recapping symptoms and personal hygiene measures should be clearly visible. Similarly, visual signs should clearly indicate the path to be followed by the patient with the surgery.

**Table 2:** Reorganisation of GP surgeries

	WHO	France	Germany	Italy	Netherlands	New Zealand	Norway	Poland	Portugal	Romania	Spain	Sweden	Switzerland	Turkey	United Kingdom	US
**Triage area**																
- Limit points of entry				x	x	x	x	x		x	x	x	x	x		x
- Place the triage station outside the facility			x				x			x		x				x
- Physical barriers in the reception area (e.g. glass, plastic window)		x	x	x	x			x		x	x					x
- Prioritize triage of patients with respiratory symptoms	x		x	x		x	x	x	x	x				x		x
- Provide screening questionnaires for all patients (including the recent onset of respiratory symptoms)	x						x	x		x	x	x	x	x		x
- Maintain a record of all patients, visitors and staff	x		x				x				x		x			
- Supply tissues, waste receptacles, alcohol-based hand sanitiser			x	x				x		x	x	x	x	x		x
- Cover nose and mouth of patients with facemasks or alternatives	x		x	x				x	x	x	x		x	x		x
- Ask patients to wash hands		x		x	x			x	x	x			x			
- Ask patients not to touch handles or open doors													x			
- Limit the number of people accompanying the patient	x	x	x	x				x		x	x		x			x
- Provide individuals accompanying the patient with face masks											x					
- Visual alerts to remind symptomatic patients to alert health professionals	x	x		x		x		x	x	x	x					x
- Visual alerts to remind patients about hand hygiene, respiratory hygiene, and cough etiquette		x	x	x	x	x		x	x	x	x		x			x
- Visual signs informing patients of the path to be followed within the surgery (e.g. COVID-19 and non-COVID-19 area)			x			x		x	x	x	x					x
**Waiting room**																
- Supply tissues, waste receptacles, alcohol-based hand sanitiser		x		x	x		x	x		x	x	x	x	x		x
- Use chairs that can be disinfected								x					x			
- Remove unnecessary objects (newspapers, unnecessary furniture, etc.)		x	x	x			x	x		x	x		x		x	
- Remember to empty the bin regularly			x					x		x	x		x			
- Organize a separate area for suspected COVID-19 patients	x	x	x		x		x	x	x	x	x		x		x	x
- Partition areas with waiting lines								x								x
- Promote distancing between patients (> 1 metre or > 6 feet apart)	x	x	x	x	x		x	x		x	x		x	x		x
- Ensure that the space is well-ventilated	x	x	x	x	x			x		x	x		x			x
- Disinfect surfaces	x	x	x	x	x		x	x	x	x	x		x	x		x
- Disinfect at least twice daily		x	x	x	x			x		x				x		
- Disinfect regularly							x	x					x			
- Waiting room organised outside the healthcare facility (e.g. patients wait in their own vehicle and are contacted by mobile phone)		x	x			x	x			x		x				x
**Examination room**																
- Organise a dedicated room			x	x	x	x	x	x	x	x	x	x	x	x	x	
- Organise a telephone consultation phone											x					
- Organise a dedicated room for children			x													
- Remember to close the door			x	x			x	x		x	x				x	
- Ask patients to wash their hands								x			x					
- Limit the number of personal items				x												
- Limit the numbers of staff providing care			x	x	x	x	x	x	x	x	x	x	x	x	x	x
- Maintain a record of all staff entering the examination room (name, time, activity, etc.)											x					
- Disinfect surfaces with which the patient comes into contact	x	x	x	x	x	x	x	x	x	x	x		x	x	x	x
- Disinfect office, computer, printer, phone, door and door handles, pens		x	x	x		x	x	x		x	x		x	x		
- Use a new paper protection sheet on the examination table for each patient	x		x	x	x	x	x	x		x	x				x	
- Manage waste safely	x		x	x	x	x	x	x		x	x		x	x	x	
- Use dedicated medical equipment	x		x	x	x	x	x	x	x	x	x	x	x	x	x	x
- Sterilise/clean equipment for patient care between patients	x	x	x	x	x	x	x	x		x	x				x	x
- Use single-use materials	x		x		x	x	x	x		x	x			x	x	

Waiting room recommendations were less detailed in New Zealand, Portugal, Sweden, and UK. In New Zealand, this was explained by the absence of waiting rooms (patients waited outside in their own vehicles). Ideally, GP surgeries should have two different waiting rooms: one for COVID-19 patients, and one for non-COVID-19 patients. If this is not possible, then the waiting room had to be partitioned with lines. Unnecessary furniture should be removed, and only hygiene supplies and washable chairs should be present. Distancing rules, sufficient ventilation and regular disinfection should also be respected.

Recommendations regarding the examination room were less detailed in France, Portugal, Sweden and USA. Ideally, GP surgeries should have a dedicated COVID-19 examination room with a closed door. Spain recommended having a telephone examination room for the remote assessment of patients. Germany recommended having special consultation hour for children, to limit virus transmission. Staff entering in the examination room should be recorded and their numbers limited. Surfaces should be disinfected, including all surfaces in contact with patients, computers, and telephones. Medical equipment should be single-use or sterilized between uses and reserved exclusively for COVID-19 patients.

### Theme 3: Reorganisation of medical examinations by GPs (Table 3)

All countries recommended that GPs should wear surgical masks during medical examinations (Table 3). They also recommended the wearing of eye protection, gloves (except France and Sweden), and gowns (except France, Germany and Sweden). Ideally, PPE should be changed between patients, but if this is not possible, its disinfection was recommended in Germany and the USA. Some countries also advised the planning of PPE use in case of shortages, whereas others, such as the UK, organised a hotline for GPs in case of shortage.

**Table 3:** Reorganisation of medical examinations performed by GPs

	WHO	France	Germany	Italy	Netherlands	New Zealand	Norway	Poland	Portugal	Romania	Spain	Sweden	Switzerland	Turkey	United Kingdom	US
**Use of appropriate personal protective equipment (PPE)**																
- Plan to optimise your facility’s supply of PPE in the event of shortages		x	x	x	x	x	x	x		x	x	x	x			x
- Use an appropriate medical mask for the medical examination	x	x	x	x	x	x	x	x	x	x	x	x	x	x	x	x
- Wear eye protection (goggles) or facial protection (face shield)	x		x	x	x	x	x	x	x	x	x		x	x	x	x
- Wear a clean, non-sterile, long-sleeved gown	x			x	x	x	x	x	x	x	x		x	x	x	x
- Use gloves	x		x	x	x	x	x	x	x	x	x		x	x	x	x
- Don't wear boots, overalls, or aprons	x									x						
- Wear aprons if gowns are not available				x												
- Use a waterproof apron if in contact with the patient’s body fluids									x		x					
- Cover shoes if they are not washable									x							
- PPE should be changed between patients	x	x			x	x		x						x	x	
- PPE should be cleaned and disinfected if re-used	x		x													x
- PPE should be changed every day, or after each suspected COVID-19 patient				x												
**Clinical examination**																
- Do not perform ENT (ear, nose and throat) examinations		x				x		x		x						
- Do not perform clinical examinations on COVID19 patients				x												
- Do not perform clinical examinations in the absence of appropriate PPE			x	x												
- Maintain a distance of at least 2 metres between yourself and the patient							x	x				x				
**Personal hygiene**																
- Hand hygiene (alcohol-based hand rub or soap and water)	x	x	x	x	x	x	x	x	x	x	x		x	x	x	x
- Hand hygiene after contact with patients or objects belonging to patients		x	x	x	x	x	x	x	x	x	x		x	x	x	x
- Refrain from touching your eyes, nose, or mouth	x		x	x		x	x	x		X			x			x
- Tie up any hair that falls onto your face			x										x			
- Shave off your beard			x					x								
- Remove jewellery			x	x			x	x		x					x	
- Remove nail varnish			x				x	x		x						
**Advice, payment**																
- Educate patients about the early recognition of symptoms, basic precautions and which healthcare facility they should go to	x		x					x		x						
- Provide clear instructions regarding home care, and how and when to access healthcare								x		x						x
- Include “personal hygiene advice” on prescriptions for COVID-19 patients		x														
- Provide COVID-19 patients with information about contact management			x							x	x					
- Develop enhanced communication skills and empathy to minimise patient anxiety	x									x	x					
- Prefer non-cash payments							x									

Italy was the only country to recommended avoiding performing clinical examinations on COVID-19 patients, particularly in the absence of appropriate PPE. France, New Zealand, Poland and Romania recommended not performing ear nose and/or throat examinations, and Norway, Poland and Sweden recommended that the doctor should stay at least 2 metres away from the patient.

Almost all countries recommended hand hygiene after patient examination and after touching objects belonging to patients. GPs should also refrain from touching their eyes, nose or mouth, but some countries also recommended tying up long hair, shaving off beards and removing jewellery and nail varnish.

France, Germany, Poland, Romania and US recommended patient education on personal hygiene and home care. Germany and Spain also recommended education about contact management. Romania and Spain also advised doctors to try to minimise patient anxiety. Norway was the only country to recommend avoiding cash payments for consultations.

### Theme 4: Reorganisation of GP surgery staff (Table 4)

Recommendations concerning the staff of GP surgeries were limited (Table 4).

**Table 4:** Reoganisation of GP surgery staff

	WHO	France	Germany	Italy	Netherlands	New Zealand	Norway	Poland	Portugal	Romania	Spain	Sweden	Switzerland	Turkey	United Kingdom	US
**Staff hygiene and equipment**																
- Hand hygiene after contact with patients or objects belonging to patients			x					x						x		
- Equip triage staff with facemasks			x					x						x		x
- Equip cleaning staff with appropriate PPE		x									x			x		
- Cleaning staff should not use a vacuum cleaner for cleaning the floor		x														
- Equip healthcare workers with an apron in cases of possible or confirmed COVID-19															x	
- Provide PPE at the entrance to the COVID-19 examination room, together with waste disposal containers											x					
**Staff management**																
- Protect vulnerable staff (pregnant, high-risk group)							x						x			
- Advise staff to check daily for any signs of illness and to notify if ill			x													x
- Isolate symptomatic staff at home			x				x	x						x		
- Authorise staff exposed to COVID-19 but without symptoms to work, with a medical mask, in cases of staff shortage			x													
- Sharing of duties, with alternation between intensive and less stressful roles														x		
- Identify healthcare personnel for the daily monitoring of patients by telephone contact								x								x
- Stop activities of voluntary non-clinical staff during the emergency period											x					
**Staff meeting**																
- Organise internal staff meeting by video-link							x									
- Designate a time to meet with your staff and educate them about COVID-19			x													x
- Inform staff about the location of specific COVID-19 areas											x					
- Support each other														x		
- Avoid eating lunch together							x									
- Bring ready-prepared food (do not prepare lunch in the lunchroom)							x									
**Extra staff – work with the local community**																
- Engage the local community to assist patients isolated at home (e.g. delivery of food)																x
- Work with local/public health organisations, and healthcare coalitions, to acquire a better understanding of the impact and spread of the outbreak in your area		x	x		x	x	x	x	x	x	x	x		x	x	x

Germany, Poland, Turkey, and the USA recommended equipping triage staff with facemasks, whereas France, Spain and Turkey recommended equipping cleaning staff with appropriate PPE. France also recommended not using a vacuum cleaner for floor cleaning.

Norway and Switzerland recommended protecting vulnerable staff, and Germany and the USA advised checking staff daily. Germany, Norway, Poland, and Turkey recommended isolating symptomatic staff at home, but Germany authorised staff exposed to COVID-19 but no symptoms to work, preconditioned they wore medical masks, in cases of staff shortage. Turkey was the only country recommending a sharing of duties at the GP practice and that staff should support each other.

Germany and the USA recommended allocating time to staff meetings, to educate staff about COVID-19. Norway advised to holding meetings by video-link and avoiding meetings over lunch.

All countries except Italy and Switzerland recommended working with local organisations to improve evaluations of the impact and spread of the outbreak in the surrounding area. Only the USA recommended collaborating with the local community to assist patients isolated at home (e.g. food delivery).

## Discussion

This is the first study to provide a multi-country overview of recommendations for the reorganisation of GP surgeries during the COVID-19 outbreak. Based on guidelines published in 15 countries, we reveal the diversity of recommendations and identify four different types of reorganisation for which recommendations have been issued: GP consultations, GP surgeries, clinical examinations performed by the GP, and GP surgery staff. The recommendations concerning GP surgery staff were limited in all countries.

### Strengths and weaknesses

This study has several strengths. Data were identified, collected and analysed by medical doctors belonging to an international GP network, with knowledge of the field of general medicine in practice. Their background as GPs made it possible to combine and compare complex guidelines in the form of “practical actions” useful for clinical practice, readily understandable by GPs and health policy-makers, of particular utility for countries in which such guidelines have yet to be developed. Moreover, this work provides an overview of recommendations published in a large panel of countries, with diverse national health systems, from the North, South, and East of Europe, but also from New Zealand and the USA, although no low-income or Asian countries were included.

This work also has some limitations. First our work focuses on comparing guidelines released at the very beginning of the pandemic. Some recommendations may have evolved quickly, varying per country (e.g., advices about the management of vulnerable patients [18], or recommendations about the diagnosis of COVID-19 in GP surgeries based on clinical symptoms [19] or on SARS-Cov2 testing [20], depending on the availability of diagnostic tests). However, as health care systems are being reorganized faster due to COVID-19 outbreak, this analysis is an opportunity to present a starting live systematic review that could then be continuously shared and updated within the primary care community. Second, comparisons between guidelines in different countries are risky, particularly during a pandemic period. Indeed, similarities or differences may be due the characteristics of the country, such as its population size, the prevalence of individuals at risk, pandemic level, or public health measures [21, 22]. However, our aim here was not to compare the successes and failures of the various countries, but to provide a global overview of guidelines relating to the organisation of GP surgeries. Indeed, the health emergency has forced health policy-makers to release guidelines rapidly, not always based on strong evidence, but justified by the precautionary principle. This has resulted in many concerns, particularly for GPs seeking answers outside their own country. Our findings should provide answers to some of the questions of GPs, although further evaluations are required to assess the level of evidence supporting the recommendations compiled here.

### Implications for policy-makers: applicability of guidelines during the pandemic

The strict application of guidelines is not an easy task in routine care. Previous studies have shown that GPs are reluctant to follow guidelines, particularly if unclear or not supported by evidence [23, 24], or if they cause major changes in routine care [23, 25]. A recent Cochrane review showed that adherence during the pandemic also depended on the complexity of the guidelines and the frequency with which they were updated [26]. However, given the huge impact of COVID-19, we assume that GPs would be receptive to well-written concise guidelines helping them to organise their care.

The application of guidelines may also depend on the characteristics of the GP surgery. Nothing has yet been published on this topic, but we hypothesise that GPs working on their own may face greater difficulties performing triage, examinations and regular cleaning, whereas GPs in group practices can share these roles. Likewise, small surgeries may find it more difficult to reorganise their practices than larger surgeries. The application of guidelines is also dependent on national health policies. For example, GPs have reported being unable to follow PPE recommendations properly [27] because of national PPE shortages.

Some GPs have also developed ingenious methods of facing the pandemic, outside the framework of official guidelines. These innovations include, delivering paper prescriptions via a window [28] and performing rapid assessments in the patient’s car [28]. Some GP-reviewers also reported having collected PPE from the population and from other healthcare professionals, or having created temporary “hot hubs” (or local COVID-19 centres) with other local GPs and the local community [28].

Further studies are required to assess the adoption of these guidelines by GPs, and their efficacy in the face of the pandemic.

### Implications for clinicians: use of digital health strategies during the pandemic

The rapid spread of COVID-19 and the possibility of GP surgeries acting as sources of contagion accelerated the adoption of digital health strategies in GP surgeries [29].

The use of telemedicine has greatly increased during the pandemic and was recommended by all 15 countries included in this study. Guidance concerning video consultations [29, 30], the various providers [31], and the performance of remote medical examinations [9], was rapidly published in several countries. Some countries also took emergency measures, such as authorising the integral reimbursement of telemedicine consultations, or authorising the use of Skype®, Facetime® and WhatsApp®, despite the need to guarantee security during such consultations [32]. Telemedicine has a number of advantages during a pandemic: shorter consultation and triage times, maintenance of contact with patients with chronic diseases and the minimisation of patient exposure to contagion [33]. However, it is too early to determine whether these practices are likely to continue after the pandemic [32, 34, 35], due to limitations, such as the risk of social inequalities (e.g. old people, the homeless, without access to Internet) [32], the risk of technical failure, and the lack of evidence that telemedicine is as effective as face-to-face consultations [36]. Further studies are required to assess the impact of telemedicine on patient care in the next few months.

Online tools for patients were also rapidly implemented during the outbreak. Educational materials were made available on government websites, to inform and educate the population. Additional interactive tools were also implemented in some countries. For example, France developed two decision support tools for self-assessments of levels of severity and susceptibility; the UK developed the NHS 111 online system for symptom checking; Spain developed “Hispabot covid-19”, a chatbot accessible by WhatsApp, for providing patients with automatic answers to their questions. Digital health solutions may be useful for alleviating the pressure on GP surgeries due to unnecessary consultations during a pandemic, but also for epidemiological surveillance by health authorities [37].

### Urgent need: involvement of GP surgeries in data collection for surveillance and clinical research

During the pandemic, the principal concern has been the reorganisation of GP surgeries for clinical care. However, it is also important to consider reorganisation for surveillance and clinical research, particularly as the computerisation of GP surgeries in recent decades [38] has greatly facilitated the collection of patient data.

The collection of data from GP surgeries would facilitate the assessment of treatments for preventing severe forms of COVID-19. However, research on these aspects is currently concentrated in hospitals: prediction models are based on data collected in hospitals [39]; and most of the clinical trials are conducted in hospitals [40]. Two months after the announcement of the pandemic by the WHO, only 23 clinical trials were registered with clinicalstrials.gov for primary care, and only 11 of these had begun to recruit patients (as of May 7^th^ 2020) [40]. GP surgeries should be reorganised and included in clinical trials assessing the efficacy of drugs for preventing severe forms of COVID-19 in primary care.

Data collection by GP surgeries is also essential for epidemiological surveillance [41]. Most cases are managed in primary care, but many countries are monitoring the pandemic through daily numbers of positive PCR-tests, deaths, calls to emergency phone centres, visits to emergency departments, and COVID-19 beds in intensive care units. However, these indicators provide a delayed and partial picture of the pandemic. A consideration of the clinical syndromes reported by GPs would facilitate the earlier and more accurate detection of peaks in disease incidence [42]. GP surgeries should therefore be reorganised to allow a real-time monitoring of the COVID-19 outbreak [43] (e.g. indicators included in electronic health records [44] or sentinel surveillance networks with selected GP practices and hospitals [45]).

## Conclusions

We provide here a multi-country overview of guidelines for reorganising GP surgeries during the beginning of the COVID-19 outbreak from 15 countries. Most of the guidelines focused on clinical care, with fewer focusing on staff management, and epidemiological surveillance and research largely neglected. Our findings should help GPs and decision-makers to adapt GP practices in public health emergencies, such as the COVID-19 outbreak, even if strong evidence is lacking for some recommendations and further evaluations are required. The impact of the reorganisation of GP surgeries on patient care should be assessed once the COVID-19 pandemic is over. Additionally, it would be helpful to propose a library of international primary care guidelines and tools to support countries in continuing guidance optimization, but also to provide historical documentation for the primary care community.

## Supplementary Information

**Additional file 1.** Materials sent to each GP-reviewer - Initial set of key recommendations collected from the World Health Organisation (WHO) [14], the Centers for Disease Control and prevention (CDC) [15] and based on health professional resources from health care facilities (GP: general practitioner).

**Additional file 2.** Guidelines used for this study.

## Data Availability

All data generated or analysed during this study are available on request to the first author RT.

## Abbreviations

GP: General Practitioner
PPE: Personal Protective Equipment
