# Supplementary material for: Reorganisation of GP surgeries during the COVID-19 outbreak: analysis of guidelines from 15 countries
Source: BMC Fam Pract. 2021 May 17;22:96. doi: 10.1186/s12875-021-01413-z (PMC8127252; doi:10.1186/s12875-021-01413-z)
Supplement: Supplementary file 1 — Additional file 1. Materials sent to each GP-reviewer - Initial set of key recommendations collected from the World Health Organisation (WHO) [14], the Centers for Disease Control and prevention (CDC) [15] and based on health professional resources from health care facilities (GP: general practitioner). [file 12875_2021_1413_MOESM1_ESM.docx]

APPENDIX 1 : Materials sent to each GP-reviewer - Initial set of key items collected from the World Health Organisation (WHO) (1), the Centers for Disease Control and prevention (CDC) (2) and based on experiences as GP from care facilities (GP: general practitioner).

| **Organisation of consultations** | | |
| --- | --- | --- |
| For COVID and non-COVID patients | Explore alternatives to face-to-face triage and visits | Consultation by phone |
|  |  | Consultation by telemedicine |
|  |  | Dedicated phone centre If yes, specify name and provider |
|  |  | Work with local/public health organisations, and healthcare coalitions to understand the impact and spread of the outbreak in your area |
| Specific for non-COVID-19 patients | For all non-COVID patients | Postpone elective procedures and non-urgent outpatient visits |
|  |  | Cancel group healthcare activities |
|  | For non-COVID patients with chronic diseases | Initiate phone contact with the patient |
|  |  | Accept the extension of drug prescriptions by pharmacists |
| Specific for COVID-19 patients |  | Remote consultation for initial diagnosis |
|  |  | Remote consultation for monitoring |
|  |  | Contact patient between days 6 and 8 for monitoring |
|  |  | Develop assessment protocols for COVID patients |
|  |  | Develop protocols for rapid triage |
|  |  | Online tools dedicated for patients If yes, specify name and goal |
|  |  | Online tools for health professionals If yes, specify name and goal |
| **Organisation of the surgery** | | |
| Appointment in GP surgery |  | Consultation with appointment (avoid consultation without appointments) |
|  |  | Provide time slot for COVID patients |
|  |  | Ensure short waiting time |
|  |  | Reschedule if development of respiratory symptoms |
| Triage area | Triage station | Limit points of entry |
|  |  | Place the triage station outside the facility |
|  |  | Provide physical barriers at the reception area (e.g. glass, plastic window) |
|  |  | Prioritise triage of patients with respiratory symptoms |
|  |  | Provide screening questionnaires for all patients (including the recent onset of respiratory symptoms) |
|  |  | Maintain a record of all patients, visitors and staff |
|  | Patients at triage area level | Supply: tissues, waste receptacles, alcohol-based hand sanitiser |
|  |  | Cover nose and mouth of patients with facemasks or alternatives |
|  |  | Ask patients to wash their hands |
|  |  | Limit the number of people accompanying the patient |
|  | Visual alerts | Remind patients with symptoms to alert health professionals |
|  |  | Remind patients about hand hygiene, respiratory hygiene, and cough etiquette |
|  |  | Signs telling patients where to go in the surgery (e.g. COVID and non-COVID areas) |
| Waiting room | Inside | Supply: tissues, waste receptacles, alcohol-based hand sanitiser |
|  |  | Remove unnecessary objects (e.g. newspapers, furniture) |
|  |  | Remember to empty the bin regularly |
|  | Area | Organise a separate area for suspected COVID-19 cases |
|  |  | Promote distancing between patients (> 1 metre or > 6 feet) |
|  |  | Remember to ventilate spaces well |
|  | Surface cleaning | Disinfect surfaces |
|  |  | Disinfect at least twice daily |
|  | Outside | Waiting room can be organised outside the healthcare facility  (e.g. Patients wait in their cars and are contacted by mobile phone) |
| Examination room | Examination room | Organise a dedicated room |
|  |  | Remember to close the door |
|  |  | Limit the numbers of staff providing care |
|  | Cleaning | Disinfect surfaces with which the patient comes into contact |
|  |  | Disinfect offices, computers, printers, phones, doors, handles, pens |
|  |  | Use a new paper protection sheet on the examination table for each patient |
|  |  | Manage waste safely |
|  | Materials | Use dedicated medical equipment |
|  |  | Sterilise patient-care equipment between patients |
|  |  | Use single-use materials |
| **Organisation of personal protective equipment (PPE)** | | |
|  | Use of appropriate personal protective equipment (PPE) | Plan to optimise your facility’s supply of PPE in the event of shortages |
|  |  | Use an appropriate medical mask during medical examinations |
|  |  | Wear eye protection (goggles) or facial protection (face shield) |
|  |  | Wear a clean, non-sterile, long-sleeved gown |
|  |  | Use gloves |
|  |  | Don't wear boots, overalls, or aprons |
|  |  | PPE should be changed between patients |
|  |  | Hand hygiene (alcohol-based hand rub or soap and water) |
|  |  | Hand hygiene after contact with patients or objects belonging to patients |
|  |  | Refrain from touching your eyes, nose, or mouth |
|  |  | Shave off your beard |
|  |  | Remove jewellery |
|  |  | Remove nail varnish |
|  |  | Do not perform ENT (ear, nose and throat) examinations |
| **Missing items** | | |
|  |  | Free text |

1. World Health Organization. Infection prevention and control during health care when covid-19 is suspected (WHO interim guidance, 19/03/2020).

2. Centers for Disease Control and Prevention. Outpatient and Ambulatory Care Settings: Responding to Community Transmission of COVID-19 in the United States (CDC guidelines, 7/04/2020) [Internet]. Centers for Disease Control and Prevention. 2020 [cited 2020 May 1]. Available from: https://www.cdc.gov/coronavirus/2019-ncov/hcp/ambulatory-care-settings.html
