# Supplementary material for: Reorganisation of GP surgeries during the COVID-19 outbreak: analysis of guidelines from 15 countries
Source: BMC Fam Pract. 2021 May 17;22:96. doi: 10.1186/s12875-021-01413-z (PMC8127252; doi:10.1186/s12875-021-01413-z)
Supplement: Supplementary file 2 — Additional file 2. Guidelines used for this study. [file 12875_2021_1413_MOESM2_ESM.docx]

APPENDIX 2: Guidelines used for this study

| **Country** | **Reference** | **Provider** | **Version used for the study**  **(DD/MM/2020)** | **Pandemic level by country**  **at the date of publication** | |
| --- | --- | --- | --- | --- | --- |
|  |  |  |  | **Cumulative number of positive cases** | **Cumulative number of deaths** |
| WHO | (1) | WHO | 19/03 | NA | NA |
| France | (2) | Public Health committee  GP college, GP teaching college | 8/04 | 83 057 | 10 887 |
|  | (3) | Health authority, GP college | 20/04 | 155 393 | 20 267 |
|  | (4) | Health ministry, GP college | 25/04 | 161 644 | 22 617 |
| Germany | (5) | National journal for GPs | 24/04 | 150 383 | 5 321 |
|  | (6) | GP association | 30/04 | 159 119 | 6 288 |
| Italy | (7) | National Scientific Society of Family Medicine | 22/04 | 106 527 | 25 969 |
|  | (8) | National Scientific Society of Family Medicine | 30/04 | 101 551 | 27 967 |
| Netherlands | (9) | GP college and representative organisation | 13/03 | 919 | 314 |
|  | (10) | GP college | 26/03 | 7 899 | 1 106 |
|  | (11) | Health authority, GP college | 23/04 | 14 897 | 1 583 |
| New Zealand | (12) | Government | 23/03 | 102 | 0 |
|  | (13) | Government | 27/03 | 368 | 0 |
| Norway | (14) | Directory of Health | 14/04 | 6 614 | 139 |
|  | (15) | Institute of Public Health | 25/04 | 7 493 | 201 |
| Poland | (16) | College of family physicians,  Polish Society for Family Medicine,  National consultation for family medicine | 15/03 | 125 | 3 |
|  | (17) | Health ministry, Chief Sanitary Inspector | 20/03 | 425 | 5 |
|  | (18) | Polish Association of Epidemiologists and Infectiology  Polish infectious diseases consultant  Chief Sanitary Inspector | 22/03 | 634 | 7 |
|  | (19) | Health ministry, Chief Sanitary Inspector | 22/03 | 634 | 7 |
| Portugal | (20) | Health authority | 23/03 | 2 060 | 23 |
|  | (21) | Health authority | 29/03 | 5 962 | 119 |
|  | (22) | GP college | 25/04 | 23 271 | 880 |
|  | (23) | Health authority | 05/05 | 25 702 | 1074 |
|  | (24) | Health authority | NA | NA | NA |
| Romania | (25) | GP respiratory group | 14/03 | 102 | 0 |
|  | (26) | GP federation | 18/03 | 260 | 0 |
|  | (27) | GP society | 06/04 | 4 057 | 157 |
|  | (28) | GP association | 06/04 | 4 057 | 157 |
| Spain | (29) | Autonomous community | 13/03/2020 | 4 209 | 120 |
|  | (30) | Health ministry, scientific societies, medical council | 17/03/2020 | 11 178 | 491 |
|  | (31) | Health Ministry, scientific societies, medical council | 14/04/2020 | 172 541 | 18 056 |
|  | (32) | Autonomous community | 21/04/2020 | 204 178 | 21 282 |
| Sweden | (33) | Public health agency | 02/04 | 5 466 | 282 |
|  | (34) | National board of health and Welfare | 07/04 | 7 693 | 591 |
| Switzerland | (35) | National organisation of physicians | 01/05 | 29 602 | 1 762 |
| Turkey | (36) | GP college | 1/04 | 15 679 | 277 |
|  | (37) | GP college | 10/04 | 47 029 | 1006 |
|  | (38) | Medical association | 11/04 | 52 167 | 1101 |
|  | (39) | Health ministry | 14/04 | 65 111 | 1403 |
| United Kingdom | (40) | National Health Service (NHS) | 10/01 | 0 | 0 |
|  | (41) | NHS | 19/03 | 3 046 | 158 |
|  | (42) | NHS | 20/03 | 3 724 | 194 |
|  | (43) | NHS | 01/04 | 26 244 | 3 095 |
|  | (44) | NHS | 06/04 | 45 040 | 6 433 |
|  | (45) | Government | 23/04 | 115 947 | 21 787 |
|  | (46) | Public Health England | 27/04 | 132 412 | 24 393 |
| US | (47) | Centers for Disease Control and Prevention | 07/04 | 333 811 | 16 191 |
|  | (48) | Centers for Disease Control and Prevention | 13/04 | 524 514 | 27 870 |
|  | (49) | American Academy of Family Physicians | 15/04 | 578 268 | 37 411 |
|  | (50) | Centers for Disease Control and Prevention | NA | NA | NA |

1. World Health Organization. Infection prevention and control during health care when covid-19 is suspected (WHO interim guidance, 19/03/2020).

2. Haut Conseil de la Santé Publique, Collège de la Médecine Générale, Collège National des Généralistes Enseignants. Avis relatif à la prise en charge à domicile ou en structure de soins des cas de COVID-19 suspectés ou confirmés (French guidelines, first version: 5/03/2020).

3. Haute Autorité de Santé, Collège de la Médecine Générale. Réponse rapide dans le cadre du COVID-19 - Assurer la continuité de la prise en charge des personnes atteintes de maladies chroniques somatiques pendant la période de confinement en ville (French guidelines, 20/04/2020).

4. Ministère des solidarités et de la santé, College de la Médecine Générale. Prise en charge en ville par les médecins de ville des patients symptomatiques en phase épidémique de Covid-19 (French guidelines, first version: 13/03/2020).

5. Ärzteblatt DÄG Redaktion Deutsches. Ambulante Versorgung: Rückkehr zum Normalbetrieb. Deutsches Ärzteblatt [Internet]. 2020 Apr 24 [cited 2020 May 5]; Available from: https://www.aerzteblatt.de/archiv/213668/Ambulante-Versorgung-Rueckkehr-zum-Normalbetrieb

6. Deutsche Gesellschaft für Allgemeinmedizin und Familienmedizin e.V. (DEGAM). ‘DEGAM S1-Handlungsempfehlung Neues Coronavirus (SARS-CoV-2) –Informationen für die hausärztliche Praxis’ (German guidelines, last version: 30/04/2020).

7. National Scientific Society of Family Medicine. Gestione del paziente con sintomi compatibili (Italian guidelines, 22/04/2020).

8. National Scientific Society of Family Medicine. Organizzazione del lavoro e assistenza in medicina generale nella fase II post covid (Italian guidelines, 30/04/2020).

9. Dutch GP college and representative organization. Practical advice organisation care for covid 19 and non covid 19 patients (Dutch guidelines, 13/03/2020).

10. Dutch GP college. Advies PBM gespecificeerd voor de huisartsenpraktijk en huisartsenpost (Dutch guidelines, 26/03/2020).

11. Dutch GP college and Health authorities. Uitvoering testen op COVID-19 bij patiënten extramuraal (Dutch guidelines, last update: 23/04/2020.

12. Ministry of Health. COVID-19 – Essential services in the health and disability system - Southlink (New Zealand guidelines, 23/03/2020) [Internet]. Ministry of Health NZ. [cited 2020 May 1]. Available from: https://www.health.govt.nz/our-work/diseases-and-conditions/covid-19-novel-coronavirus/covid-19-current-situation/covid-19-essential-services-health-and-disability-system

13. Ministry of Health. Personal protective equipment use in health care (New Zealand guidelines, 27/03/2020) [Internet]. Ministry of Health NZ. [cited 2020 May 1]. Available from: https://www.health.govt.nz/our-work/diseases-and-conditions/covid-19-novel-coronavirus/covid-19-novel-coronavirus-information-specific-audiences/covid-19-advice-workers-including-personal-protective-equipment/personal-protective-equipment-use-health-care

14. Norwegian Directory of Health. Coronavirus - decisions and recommendations - primary health care (Norway guidelines, last update: 14/04/2020).

15. Norwegian Institute of Public Health. GPs and ER - Infection control against Covid-19 (Norway guidelines, first version: 8/02/2020).

16. The College of Family Physicians in Poland, Polish society of family medicine, National consultant for family medicine. Wskazówki postępowania dla lekarzy POZ wzwiązku z zagrożeniem epidemią CoViD-19 Wybrane aspekty organizacyjne stan na (Polish guidelines, 15/03/2020).

17. Ministry of health, Chief Sanitary Inspector. Algorithm of proceeding in case of suspicion of CoViD-19 (Polish guidelines, 20/03/2020).

18. Polish Association of Epidemiologists and Infectiology, Polish consultant of infectious diseases, Chief Sanitary Inspector. The recommendations of the Polish Association of Epidemiologists and Infectiologists, Polish consultant of infectious diseases and the Chief Sanitary Inspector regarding the proceeding with SARS-CoV-2 positive patients who do not require hospitalization (Polish guidelines, 22/03/2020).

19. Ministry of health, Chief Sanitary Inspector. Zalecenia dla pacjenta z dodatnim wynikiem badania w kierunku koronawirusa ze wskazaniem do izolacji w warunkach domowych (Polish guidelines, 22/03/2020).

20. Direccao Geral de Saude. COVID-19: Fase de mitigacao. Abordagem do Doente com Suspeita ou Infeção por SARS-CoV-2 (Portuguese guidelines, 23/03/2020).

21. Direccao Geral de Saude. Prevenção e Controlo de Infeção por SARS-CoV-2 (COVID-19): Equipamentos de Proteção Individual (EPI) (Portuguese guidelines, 29/03/2020).

22. Colegio da Especialidade de Medicina Geral e Familiar. Recomendação do Colégio de MGF sobre organização de ‘mini-equipas’ exclusivamente destinadas ao serviço em ADC nos CSP (Portuguese guidelines, 25/04/2020).

23. Administracao Regional de Saude do Norte. Reestruturacao de servicos – Em estado de calamidade – ACES E Unidades Funcionais (Portuguese guidelines, 05/05/2020).

24. Direccao Geral de Saude. Plano Nacional de Preparacao e Resposta a Doenca por Novo Coronavirus (COVID-19) (Portuguese guidelines, 2020).

25. Grupul RespiRO. Recomandări privind organizarea activității în cabinetele de medicina familiei, în contextul epidemiologic actual al infecției COVID 19 (Romanian guidelines, 14/03/2020).

26. Federația Națională a Patronatelor Medicilor de Familie. Recomandări pentru cabinetele de medicina familiei pe perioada stării de urgenţă (Romanian guidelines, 18/03/2020).

27. Societatea Nationala de Medicina Familiei/Medicina Generala. Managementul pacientilor simptomatici la nivelul medicului de familie, in etapa epidemica a COVID-19 (Romanian guidelines, 06/04/2020).

28. Asociatia Medicilor de Familie din Bucuresti si judetul Ilfov. Infografic – Covid-19: Consultații la distanță (Romanian guidelines, 06/04/2020).

29. Servicio Andaluz de Salud. BOJA Extraordinario núm. 5 - Viernes, 13 de Marzo de 2020. Sevilla: Consejería de Salud y Familias (Spanish guidelines, 13/03/2020).

30. Ministerio de Sanidad, Consumo y Bienestar Social, Gobierno de España. Manejo en atención primary del COVID-19 (Spanish guidelines, 17/03/2020).

31. Ministerio de Sanidad, Consumo y Bienestar Socia, Gobierno de España. Prevención y control de la infección en el manejo de pacientes con COVID-19 (Spanish guidelines, 14/04/2020).

32. Servicio de Salud del Principado de Asturias. Manejo en Atención Primaria de COVID-19 en Asturias. Oviedo: Conserjería de Salud (Spanish guidelines, 21/04/2020).

33. Folkhälsomyndigheten. Rekommendationer för handläggning och val av skyddsåtgärder mot covid-19 inom vård och omsorg (Swedish guidelines, first version 30/03/2020).

34. Socialstyrelsen. Triage/flöden och arbetssätt vid covid-19 (Swedish guidelines, 07/04/2020).

35. National organization of physicians (FMH). COVID-19: Schutzkonzept der FMH zum Betrieb von Arztpraxen (Swiss guidelines, 1st version 27/04/2020).

36. Turkish Association of Family Physicians (TAHUD). COVID-19 Management in Family Health Centers (Turkish guidelines, 04/01/2020).

37. Turkish Association of Family Physicians (TAHUD). Recommendations for Protection of Physicial and Mental Health for the Family Health Center Staff during COVID-19 Outbreak (Turkish guidelines - 10/04/2020).

38. Turkish Medical Association. Guidance for Family Health Centers during COVID-19 Outbreak (Turkish guidelines (Turskish guidelines, 11/04/2020).

39. Ministry of Health. Guidance to COVID-19 (Turkish guidelines, first version: 24/01/2020).

40. National Health System. Putting on personal protective equipment for non-aerosol generating procedures (UK guidelines, 10/01/2020).

41. National Health System. Next steps on general practice response to COVID-19: 19 March 2020 (UK guidelines, 19/03/2020).

42. National Health System. Guidance on supply and use of PPE (UK guidelines, 20/03/2020).

43. National Health System. Advice on how to establish a remote ‘total triage’ model in general practice using online consultations (UK guidelines, 01/04/2020).

44. National Health System. Guidance and standard operating procedure: General Practice in the Context of Coronavirus (COVID-19) version 2.1 (UK guidelines, 6/04/2020).

45. UK government. Guidance: Coronavirus: How to help safely (UK guidelines, 23/04/2020).

46. Public Health England. COVID-19: Infection prevention and control guidance (UK guidelines, 27/04/2020).

47. Centers for Disease Control and Prevention. Outpatient and Ambulatory Care Settings: Responding to Community Transmission of COVID-19 in the United States (CDC guidelines, 7/04/2020) [Internet]. Centers for Disease Control and Prevention. 2020 [cited 2020 May 1]. Available from: https://www.cdc.gov/coronavirus/2019-ncov/hcp/ambulatory-care-settings.html

48. Centers for Disease Control and Prevention. Interim Infection Prevention and Control Recommendations for Patients with Suspected or Confirmed Coronavirus Disease 2019 (COVID-19) in Healthcare Settings (CDC guidelines, updated: 13/04/2020) [Internet]. Centers for Disease Control and Prevention. 2020 [cited 2020 May 1]. Available from: https://www.cdc.gov/coronavirus/2019-ncov/hcp/infection-control-recommendations.html

49. American academy of family physicians (AAFP). COVID-19: Guidance for Family Physicians on Preventive and Non-Urgent Care (American guidelines, 15/04/2020).

50. Centers for Disease Control and Prevention. Healthcare Facilities: Preparing for Community Transmission (CDC guidelines, 29/02/2020) [Internet]. Centers for Disease Control and Prevention. 2020 [cited 2020 May 1]. Available from: https://www.cdc.gov/coronavirus/2019-ncov/hcp/guidance-hcf.html
